# Comparative Analysis of Skeletal Muscle DNA Methylation and Transcriptome of the Chicken Embryo at Different Developmental Stages

**DOI:** 10.3389/fphys.2021.697121

**Published:** 2021-07-02

**Authors:** Jinshan Ran, Jingjing Li, Lingqian Yin, Donghao Zhang, Chunlin Yu, Huarui Du, Xiaosong Jiang, Chaowu Yang, Yiping Liu

**Affiliations:** ^1^Farm Animal Genetic Resources Exploration and Innovation Key Laboratory of Sichuan Province, Sichuan Agricultural University, Chengdu, China; ^2^Animal Breeding and Genetics Key Laboratory of Sichuan Province, Chengdu, China

**Keywords:** DNA methylation, transcriptome, muscle development, chicken, embryonic

## Abstract

DNA methylation is a key epigenetic mechanism involved in embryonic muscle development and plays an important role in early muscle development. In this study, we sought to investigate the effects of genome-wide DNA methylation by combining the expression profiles of the chicken embryonic muscle. Genome-wide DNA methylation maps and transcriptomes of muscle tissues collected from different embryonic development points (E7, E11, E17, and D1) were used for whole-genome bisulfite sequencing (WGBS) and RNA sequencing, respectively. We found that the differentially methylated genes (DMGs) were significantly associated with muscle organ development, regulation of skeletal muscle satellite cell proliferation, and actin filament depolymerization. Furthermore, genes *TBX1*, *MEF2D*, *SPEG*, *CFL2*, and *TWF2* were strongly correlated with the methylation-caused expression switch. Therefore, we chose the *CFL2* gene to explore its function in skeletal muscle satellite cells, and the *in vitro* experiments showed that *CFL2* acts as a negative regulator of chicken skeletal muscle satellite cell proliferation and can induce cell apoptosis. These results provide valuable data for future genome and epigenome studies of chicken skeletal muscle and may help reveal the molecular mechanisms of potential economic traits.

## Introduction

Skeletal muscle, as the main component of meat, is one of the most important agricultural animal economic traits. It is developed from myogenic precursor cells called myoblasts. Myoblasts proliferate and then differentiate into myotubes, and finally, myotubes differentiate into muscle fibers (Picard et al., 2002). It is generally believed that the proliferation of muscle fibers in animals, especially birds and mammals, mainly occurs during the embryonic period, and the number of muscle fibers is fixed and remains unchanged after birth (Yin et al., 2014). In birds and mammals, postnatal muscle growth is mainly achieved by skeletal muscle satellite cells fusing with existing fibers to cause muscle hypertrophy. These satellite cells have the potential of stem cells. In newborn animals, they actively proliferate and supplement the nuclei of existing muscle fibers. In adult animals, their mitosis remains stationary and becomes active only when skeletal muscles are injured or damaged (Moss and Leblond, 1971). A comprehensive understanding of the genetic basis of skeletal muscle performance is essential for animal breeding and human biomedical research related to muscles.

Epigenetics refers to the fact that there is a heritable change in gene expression with no change in the base sequence of DNA. Epigenetic mechanisms include DNA methylation, histone modifications, and microRNAs (Goldberg et al., 2007). Among them, DNA methylation is involved in almost all biological functions, including embryonic development, cell proliferation and differentiation, genomic imprinting, and disease occurrence (Chiappinelli et al., 2015). A crucial role for DNA methylation in muscle development has been reported in humans (Miyata et al., 2015), pig (Yang et al., 2017), rabbits (Huszar, 1972), and chickens (Zhang et al., 2017). DNA methylation in the promoter region and gene body can stably change the gene expression, and this process has important effects on the development and tissue-specific gene expression (Jones, 2012; Yang et al., 2014). Genome-wide methylation and transcriptome association analysis is a commonly used method to study methylation function. Multi-omics association analysis can clarify important signal regulation pathways at multiple levels, and it is also an effective means for large-scale identification of new biological regulatory networks.

In this study, we performed DNA methylome profiling of chicken embryo skeletal muscle in Jiuyuan Black chicken using whole genome bisulfite sequencing (WGBS). A comprehensive analysis of genome-wide DNA methylation and transcriptome was performed to reveal the way DNA methylation can regulate muscle performance by affecting gene expression. We observed that gene expression is negatively correlated with DNA methylation in the promoter regions in chicken. We also screened differentially methylated genes and differentially expressed genes, and explored its role in chicken satellite cells. Our research provides an empirical basis in order to further explore the molecular regulation mechanism of muscle development in the early stage of poultry.

## Materials and Methods

### Ethics Approval

All experimental operations were approved by the Animal Ethics Committee of Sichuan Agricultural University, and the approved number was B20171901-1. Relevant guidelines and regulations were followed while performing all the methods.

### Tissue Sampling

The half-sibling fertilized eggs of Jiuyuan black chicken were incubated in the same condition (Temperature 37.8°C, relative humidity 60%, maintain ventilation). The leg muscle and blood were collected at E7, E11, E17, and D1. After sex determination by PCR, only samples identified as male were kept for the next experiments. A total of 12 embryonic chickens were used in the study to form four groups. Each group included three individuals as biological replicates.

### DNA and RNA Extraction

The skeletal muscle derived from leg tissues of three embryos in different stages, E7, E11, and E17 (days 7, 11, and 17), and one newborn (D1) was stored in liquid nitrogen immediately after collection and DNA isolated from each aliquot using the QIAamp DNA Mini Kit (QIAGEN, Germany) following the recommended instruction. DNA concentration and quality were evaluated using the Qubit^®^ DNA Assay Kit in Qubit^®^ 2.0 Flurometer (Life Technologies, Carlsbad, CA, United States). Total RNA was isolated using the TRIzol (TAKARA, Dalian, China) reagent according to the manufacturer’s instruction. RNA was reverse transcribed by the TAKARA PrimeScriptTM RT reagent kit (TAKARA) according to the manufacturer’s instruction. The integrity of RNA was measured using agarose gel electrophoresis, and the purity was evaluated by NanoDrop 2000 spectrophotometer. Only qualified RNA samples were considered acceptable for sequencing.

### Genome-Wide DNA Methylation Profiling

Genome-wide DNA methylation profiling of *Gallus gallus* skeletal muscle from four developmental stages was established using an Illumina Hiseq 2500 platform, and 150 bp paired-end reads were generated according to the manufacturer’s standard procedure. In brief, genomic DNA was fragmented by sonication to 200–300 bp with Covaris S220, followed by end repair and adenylation. Then, these DNA fragments were treated with bisulfite using the EZ DNA Methylation Kit (Zymo Research, Irvine, CA, United States), before PCR amplification using KAPA HiFi HotStart Uracil + ReadyMix (2X). The resulting DNA products were used for library preparation and subsequent sequencing, and more than 140 million raw reads were produced in each sample. Raw image intensities were scanned by the iScan SQ scanner (iScan System, Illumina, United States) and processed by the Genome Studio software (Illumina, United States). The methylated rate of cytosine (Mc) was calculated as β value in designed windows bins (bin size is 10 kb), and the β value varied from 0 (completely unmethylated) to 1 (completely methylated). Overall, more than 4,100 Mb cytosine sites were measured in all of the samples. The β value was corrected according to Lister et al. (2013) studies to mitigate false positives from bisulfite non-conversion rate.

### Differential Methylation Analysis

Differentially methylated regions (DMRs) were identified by a moderated dispersion shrinkage method of the DSS software (Park and Wu, 2016), which combines spatial correlation, read depth of the Cytosine sites, and variance among biological replicates to precisely detect DMRs in improving statistical tests. Genomic feature of DMRs was defined based on gene coordinates on the *Gallus gallus* genome annotation file,^1^ and the intervals between upstream 1,000 bp from transcription start site (TSS) to downstream from 200 bp were considered as promoter regions. The DMRs with a greater absolute value of statistical scores between two groups have a more probability of methylation difference.

### RNA-Seq Data Preparation and Sequencing

Total RNA was extracted from the collected leg tissues as described above (with three replicates per group) by using the TRIzol reagent (Catalog No.15596-026, Invitrogen, Carlsbad, CA, United States) in accordance with the protocol. A total amount of 3 μg of RNA per sample was used as input material for the RNA sample preparations. RNA integrity was assessed using the RNA Nano 6000 Assay Kit of the Bioanalyzer 2100 system (Agilent Technologies, Santa Clara, CA, United States) with the qualified standard RIN value of >7. After rRNA depletion, second-strand cDNA synthesis, RNase H digestion, and adding adaptor in the 3′ end, the cDNA library for sequencing was constructed by PCR amplification. Then, the cDNA library qualification was detected and sequencing was performed on an Illumina Hiseq platform, and 150 bp paired-end (PE150) reads were generated.

### Transcriptome Assembly

The clean data were mapped to the *Gallus gallus* genome^2^ using the read aligner HISAT2 (version 2.0.4). Next, the transcriptome was assembled by the StringTie (version 1.3.1) on the basis of the reads mapped to the chicken genome. Annotated gtf files for each samples were produced and their raw counts information for each gene was extracted by using the python script prepDE.py (Pertea et al., 2016).

### Differential Expression Analysis

The raw count data from the StringTie output was normalized through the median of ratios method in the DESeq2 (Love et al., 2014) package in R, to exclude the bias from sequencing depth and RNA composition. Normalized counts were used for differential expression analysis and the genes in comparison with an adjusted *p*-value < 0.05 were assigned as differentially expressed (DE).

### Time-Series Expression Profile Analysis

The genes that were DE in at least one adjacent time point comparison (E7 vs. E11, E11 vs. E17, and E17 vs. D1) were used to perform the expression pattern analysis by STEM (version 1.3.11). The expression levels of transcripts were normalized [log_2_ (E7/E7), log_2_ (E11/E7), log_2_ (E17/E7), and log_2_ (D1/E7)] before being co-expressionally clustered. There are many designed modal profiles in the STEM software.^3^ After inputting these gene expression data, genes were assigned to the model profiles that most closely represented their expression patterns as determined by the correlation coefficient (*r* > 0.7). The profiles with *p* < 0.05 were identified as significant temporal expression profiles, which obviously responded to the embryonic development and muscle tissue processing.

### Gene Functional Enrichment Analysis

Biological process in gene ontology (GO) terms was conducted via the GO Biological Process 2018 data source in Enrichr (Kuleshov et al., 2016), which calculates the combined score by taking the log of the *p*-value from the Fisher exact test and multiplying that by the z-score of the deviation from the expected rank.

### Protein Interaction Network of Integrated Genes

We used the STRING database (v11.0) (Damian et al., 2017) to construct and screen for a protein-protein interaction (PPI) network that contained differentially methylated and expressed genes with obvious functional enrichment related to muscle development. We only retained edges of the network that meet the following parameters: confidence score >0.8 and combined score >0.8. Cytoscape (v3.6.0)^4^ was used to visualize interactions for the gene-gene pair input, including their strength of interaction reflected by the thickness of the line.

### Cell Culture

Following the procedures described in previous studies, SMSCs were isolated and cultured from the pectoralis major of 3-day-old Jiuyuan black chicken. The pectoral muscle was collected and shredded to release cells with 0.1% collagenase I (Sigma Chemical Co., St. Louis, MO, United States) and 0.25% trypsin (Gibco, Grand Island, NY, United States). The satellite cells were isolated from the cell suspension by filtration and differential adhesion. Then, the 10% growth medium [GM: Dulbecco’s modified Eagle medium (DMEM) (Gibco), +10% fetal bovine serum (Gibco), +0.2% penicillin/streptomycin (Invitrogen, Carlsbad, CA, United States)] was added to culture the isolated satellite cells. When the cell density reached 70–80% in the growth medium, the cells were cultured in the differentiation medium [DM: DMEM + 2% horse serum (Gibco)] instead, which is used to induce differentiation. The cells were cultured in a constant temperature and humidity cell incubator (Thermo Scientific, San Jose, CA, United States) (5% CO2 humid atmosphere, 37°C), and the medium was changed daily.

### Cell Counting Kit-8 (CK-8) and 5-Ethynyl-2-Deoxyuridine (EdU) Assay

SMSCs were seeded in 96-well plates and transfected with siRNA, negative control. A Cell Counting Kit-8 kit (Multisciences, Hangzhou, China) was used to detect cell proliferative activity according to the manufacturer’s instructions. Then, 10 μl of CCK-8 Reagent was added to each well cells at 12, 24, 36, and 48 h after cell transfection, and the cells were cultured in a constant temperature incubator (37°C, 5% CO2) for 2 h. The optical density (OD) of each sample at 450 nm was measured by a Thermo Scientific^TM^ Varioskan LUX. For the EdU assay, the proliferation state of muscle cells was performed with the Cell-Light^TM^ EdU kit according to the manufacturer’s instructions. The quantities of EdU-positive cells were calculated using a fluorescence microscope (Olympus, Tokyo, Japan).

### Cell Apoptosis Assay

Cells were stained with the annexin V-fluorescein isothiocyanate (FITC)/propidium iodide (PI) kit (Multisciences). The detailed procedures refer to the manufacturer’s instruction and previous research (Li et al., 2018). Cell apoptosis was analyzed by using flow cytometry (Becton Dickinson).

### Real-Time Quantitative PCR (qRT-PCR) and Western Blot Analysis

qRT-PCR analysis was performed in 10 μl reaction volumes containing 1 μl of cDNA, 0.5 μl of forward and reverse primers, 5 μl of TB Green^TM^ premix (Takara), and 3 μl of DNase/RNase Free deionized water (Tiangen, Beijing, China). The relative expressions of related genes were calculated by the 2^–ΔΔ*Ct*^ method, and three biological replicates were performed on each sample. The proteins were extracted on ice using commercial protein extraction kits (BestBio Biotech Co., Ltd., Shanghai, China) and adjusted to the same concentration, then placed at 95°C to denature for 5 min. The total volume of each well was 20 μl, including 16 μl of protein sample and 4 μl of reducing loading buffer (4:1). The steps and details of the Western blot analysis experiment are described in depth by Liu et al. (2019). The antibodies were diluted according to the manufacturer’s instructions as follows: anti-Bcl2 (Santa Cruz Biotechnology, Santa Cruz, CA, United States), anti-Caspase 3 (Biorbyt, Cambridge, United Kingdom), anti-CDK2 (ABclonal Technology, Wuhan, China), anti-PCNA (ABclonal), and anti-CLF2 (ABclonal, Wuhan, China). The last, anti-β-actin (ZenBio, Chengdu, China; 1:2,000), was used as a loading control.

## Results

### Genome-Wide DNA Methylation Patterns in Four Developmental Stages

**FIGURE 1:**
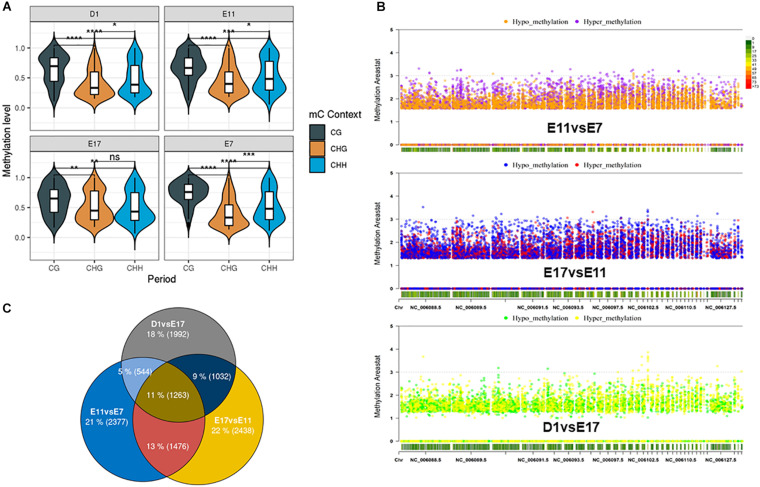
DNA methylation profiles of leg muscle samples in four stages of chicken embryo. **(A)** Genome wide DNA methylation in three contexts of cytosine, *, **, ***, and **** mean *p*-value < 0.05, 0.01, 0.001, and 0.0001, respectively. D1: First day after birth; E7, E11, and E17 represent the 7th, 11th, and 17th day of embryonic stage, respectively. **(B)** Hypo- and hypermethylated regions across chromosomes in the comparison between two periods. **(C)** Amount and overlap of DMRs among the three compared groups.

**FIGURE 2:**
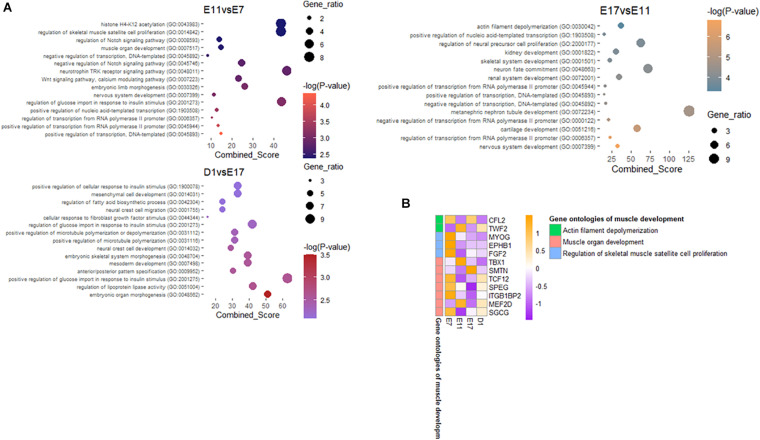
Functional enrichment of DMRs. **(A)** Gene ontology terms of differentially methylated genes in E11 vs. E7, E17 vs. E11, and D1 vs. E17 comparisons, respectively. Combined score is computed by taking the log of the *p*-value from the Fisher exact test and multiplying that by the *z*-score of the deviation from the expected rank. **(B)** The methylation levels of the promoter regions of the genes related to muscle development.

Our objective was to determine DNA methylation and transcriptomic dynamics through the embryonic process that controls skeletal myogenesis in chicken tissues. From the genome wide DNA methylation landscape, cytosine with CG context accounted for the largest proportion in all developmental periods; more than 93% of methylated cytosines were adjacent to the guanines (Supplementary Figure 1). We found that the average methylation level of the three contexts is almost the same in the functional regions of the genome at the four developmental periods. In the CG context, the lowest methylation levels occurred in the promoter and utr 5 regions, while CHG and CHH contexts have the highest methylation levels in these two regions (Supplementary Figure 2). The methylation levels of cytosines in three different contexts also had preferences that CG context showed higher DNA methylation levels among the four groups; while methylation levels in CHG and CHH contexts were slightly lower (Figure 1A). In addition, the difference in the methylation levels between CG, CHH, and CHG contexts was extremely significant during E7, while during E17, there was no significant difference in the methylation levels between CHH and CHG contexts. Methylation profiles on the chicken genome were divided into specified and non-overlapped regions by the DSS software [13], the differentially methylated values were corrected by the Areastat score. The amount of DMRs were proportional with the size of the chromosomes, and there were more hypomethylated regions in E11 and E17 periods compared to E7 and D1 (Figure 1B). Based on the DMRs categorized by genomic elements, most of the DMRs were distributed on intronic regions and they were less than 200 bp on average (Supplementary Figures 3, 4). After assigning the annotation information to the DMRs, 5,660, 6,209, and 4,831 differentially methylated genes were obtained in E11 vs. E7, E17 vs. E11, and D1 vs. E17 groups, respectively (Figure 1C). To further understand the potential functions of genes that have differentially methylated regions compared to the nearing periods, gene ontology (GO) terms enrichment was conducted using DMRs contained genes (Figure 2A). There were muscle organ development (GO: 0007517) and regulation of skeletal muscle satellite cell proliferation (GO: 0014842) enriched by differentially methylated genes in E11 vs. E7 groups, and actin filament depolymerization (GO: 0030042) was an enriched GO term in E17 vs. E11 comparison. We focused on the genes that participate in the muscle development related GO terms and found their methylation levels in the promoter regions, which showed opposite trends with their expressional mode (Figure 2B). Most of the genes that were included in the biological process we described above had a lower promoter methylation level, indicating that their activated transcription potential may trigger the active muscle development in the middle and late stages of the egg embryo.

### Continuous Expressional Activation of Muscle Development Genes Linked With Promoter Methylation

**FIGURE 3:**
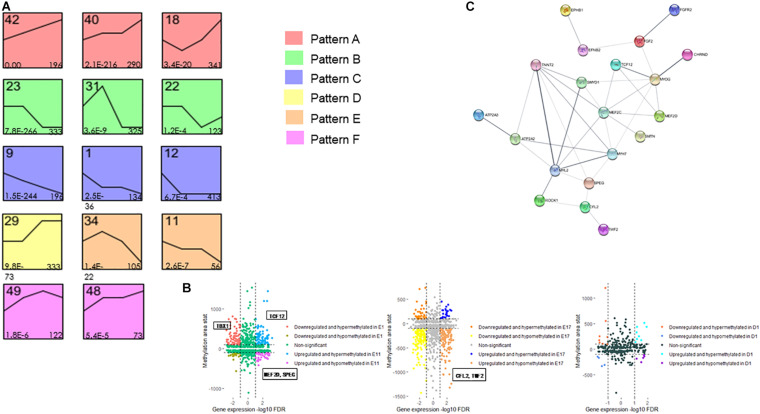
Transcription of muscle development related genes is correlated with promoter DNA methylation. **(A)** Significant expression profiles (*p* < 0.05) of differentially expressed genes clustered via the STEM software during embryonic stages. The numbers in the top left corner of boxes are profile serial numbers, those in the bottom left corner are *p* values, and those in the bottom right corner are numbers of genes contained in profiles. Six general patterns were determined (patterns A–F) based on their similar expression features. **(B)** Quadrant plot showing promoter methylation and expression levels of the corresponding genes. The vertical dotted lines indicate a threshold of the adjusted *p*-value (FDR) equals 0.05, and parallel dotted lines show a threshold of the absolute methylation AreaStat equals 100. **(C)** The protein-protein interaction (PPI) network among both differentially methylated and expressed genes, and line thickness shows their strength of interaction.

To identify the expression patterns during embryonic development, a Short Time-series Expression Miner (STEM) was conducted to cluster transcripts based on expressional trends during the time course. We applied all differentially expressed genes in the DESeq2 analysis and 12,473 genes were input to the STEM analysis. In total, 50 model profiles were detected and 14 expression profiles were specified with sufficient gene numbers and significant co-expression trends (Figure 3A). The significant expression patterns can be categorized into six feature patterns (Figure 3A) based on their continuous changes compared to the starting time point (E7). Intriguingly, we found that profile 48 always performed an obvious enrichment in muscle-related GO terms compared to other expression profiles (Table 1). Then, we integrated the methylation levels of genes corresponding to their expression profiles and found that their positive and negative relationships in genes are both differentially methylated and expressed (Figure 3B). Notably, genes *TBX1*, *TCF12*, *MEF2D*, *SPEG*, *CFL2*, and *TWF2* were strongly correlated with the methylation-caused expression switch and their functions in embryonic muscle formation should be investigated further. Moreover, to understand the protein interactions between the genes of interest, a protein-protein interaction (PPI) network was constructed and their strength of interaction, which was reflected through the line thickness, was showed with other actively interacted genes (Figure 3C and Supplementary Table 1).

**TABLE 1:** Expression profile 48 enriched GO terms.

**Category ID**	**Category_name**	**Cluster enrichment *p*-value**	**Gene included**
GO:0071340	skeletal muscle acetylcholine-gated channel clustering	0.00041	*FZD9,RER1,FNTA*
GO:0060538	skeletal muscle organ development	0.04	*SMYD1,MEF2C,CHRND,FBXO22*
GO:0007519	skeletal muscle tissue development	0.03	*SMYD1,MEF2C,CHRND,FBXO22*
GO:0055001	muscle cell development	0.03	*MEF2C,ATP2A2,FBXO22*
GO:0042692	muscle cell differentiation	0.04	*SMYD1,MEF2C,ATP2A2,FBXO22*
GO:0042693	muscle cell fate commitment	0.02	*MEF2C*
GO:0042694	muscle cell fate specification	0.02	*MYL2*
GO:0014812	muscle cell migration	0.04	*MEF2C,ROCK1*

### CFL2 Inhibits Chicken Skeletal Muscle Satellite Cells Proliferation

**FIGURE 4:**
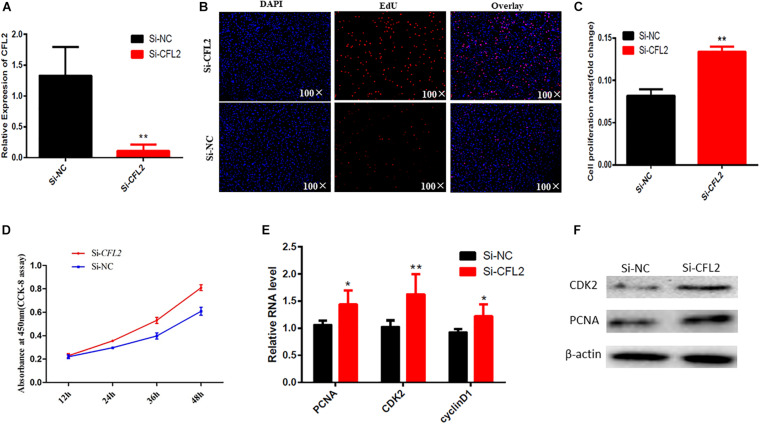
*CFL2* regulates chicken skeletal muscle satellite cells proliferation. **(A)** Detection of *CFL2* mRNA expression after transfection of *CFL2* siRNA. **(B)** EdU staining-positive muscle cells were detected by the EdU kit after knockdown *CFL2*. **(C)** Cell proliferation rate after knockdown of *CFL2*. **(D)** Cell proliferation status was detected within 48 h at 450 nm with CCK-8 reagent after knockdown of *CFL2*. **(E)** Cell proliferation-related genes (*PCNA*, *CDK2*, and *cyclinD1*) mRNA expression level was detected by qRT-PCR after the knockdown of *CFL2*. **(F)** Cell proliferation-related genes (PCNA and CDK2) protein expression level was detected by Western blot. Data are presented as mean ± SEM; *p < 0.05 and **p < 0.01.

In order to reveal the function of *CFL2* in chicken skeletal muscle satellite cells, we transfected the *CFL2* siRNA into chicken SMSCs to assess its effect on cell proliferation and apoptosis. After the knockdown of *CFL2* with siRNA, the mRNA expression level of *CFL2* could be inhibited significantly (Figure 4A). The quantities of EdU staining-positive cells were increased after the knockdown of *CFL2* (Figures 4B,C). CCK-8 assay can detect cell viability, and the results showed that SMSCs proliferation was significantly promoted following *CFL2* knockdown (Figure 4D). In parallel, the expression of cell proliferation marker genes *PCNA*, *CDK2*, and cyclinD1 were detected. qRT-PCR results showed that the knockdown of *CFL2* increased the mRNA expression levels of *PCNA*, *CDK2*, and *cyclinD1* (Figure 4E). Furthermore, the Western blot results showed that the expression of PCNA and CDK2 was promoted by transfection with *CFL2* siRNA (Figure 4F). Collectively, these results demonstrate that *CFL2* could inhibit chicken skeletal muscle satellite cells proliferation.

### CFL2 Promotes Chicken Skeletal Muscle Satellite Cells Apoptosis

**FIGURE 5:**
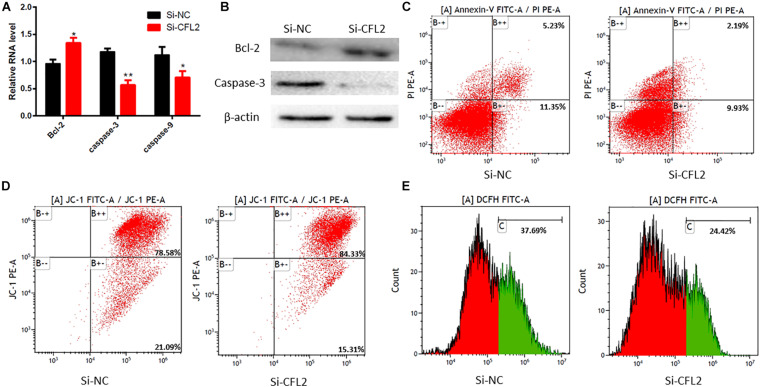
*CFL2* regulates chicken skeletal muscle satellite cells apoptosis. **(A)** Cell apoptosis-related genes (*Bcl2*, *Caspase 3*, and *Caspase 9*) mRNA expression level was detected by qRT-PCR after the knockdown of *CFL2*. **(B)** Cell apoptosis-related genes (Bcl2 and Caspase 3) protein expression level was detected by Western blot. **(C)** Apoptotic muscle cells were detected by annexin V-FITC/PI-staining flow cytometry after the knockdown of *CFL2*. **(D)** Mitochondrial transmembrane potential (ΔΨm) was detected by annexin V-FITC/PI-staining flow cytometry after knockdown of *CFL2*. **(E)** Reactive oxygen species (ROS) content was detected by annexin V-FITC/PI-staining flow cytometry after knockdown of *CFL2*. Data are presented as mean ± SEM; *p < 0.05 and **p < 0.01.

We also detected some cell survival genes *Bcl-2*, *caspase3*, and *caspase9* mRNA level by qRT-PCR. Compared with the control group, the expression levels of *caspase3* and *caspase9* in the *CFL2* SiRNA group were decreased, but the *Bcl-2* expression level was increased (Figure 5A). The corresponding protein levels of these genes also showed the same changes like their mRNA level (Figure 5B). Additionally, the number of apoptotic cells in the *CFL2* siRNA group was decreased compared with the control group (Figure 5C). We also found that the mitochondrial transmembrane potential (ΔΨm) and reactive oxygen species (ROS) content decreased with the knockdown of *CFL2* (Figures 5D,E). All of these results indicate that *CFL2* promotes chicken skeletal muscle satellite cells apoptosis.

## Discussion

DNA methylation contributes substantially to phenotypic variations in aging (Bell et al., 2012), obesity (Wang et al., 2010), and body size (Relton et al., 2012). However, the regulatory mechanism of DNA methylation affecting chicken embryo skeletal muscle performance is still unclear. As a tissue of major economic importance in meat-producing animals, skeletal muscle plays important roles in initiating movements, supporting respiration, and maintaining homeostasis (Ge et al., 2014). In addition, chicken provides a unique model to perform embryological research due to the accessibility of its egg. Here, we use the Jiuyuan black chicken to explore the development of skeletal muscle in the embryonic period of chickens. We performed whole genome bisulfite sequencing and RNA-seq to systematically explore the prenatal DNA methylation landscape during chicken muscle development. We mainly focus on the systematic study of the four different embryonic stages of E7, E11, E17, and D1 to find the factors that affect the development of skeletal muscle.

Different CpG contexts (CG, CHG, and CHH) at the four developmental stages have similar DNA methylation levels and proportions, with the highest CG content and extremely low CHG and CHH content. These results were in accordance with findings in other species (Laurent et al., 2010; Sati et al., 2012). It has been well documented that gene-body exhibits higher methylation than the 5′ flanking regions and promoter methylation negatively correlates with gene expression (Law and Jacobsen, 2010; Zemach et al., 2010). For the gene body regions, we did not observe a higher methylation level in exons than in introns in chickens, which is in contrast to a previous research (Hu et al., 2013). Gene body methylation and expression levels apparently have a complex relationship, it has been demonstrated that CGIs could influence local chromatin structure (Deaton and Bird, 2011). In the present study, we found a large number of methylated CGIs in the intragenic and intergenic regions. These CGIs were proven to have the characteristics of functional promoters and the methylation of intragenic CGIs played a crucial role in regulating alternative promoters (Illingworth et al., 2010; Maunakea et al., 2010).

Next, we comprehensively compared the methylation levels of genes among four different developmental stages. There were more hypomethylated regions in E11 and E17 periods compared to E7 and D1, which may be responsible for their different speeds in muscle development. After assigning the annotation information to the DMRs, 5,660, 6,209, and 4,831 differentially methylated genes were obtained in E11 vs. E7, E17 vs. E11, and D1 vs. E17 groups, respectively. In chickens, Myofiber ontogenesis begins with the appearance of two successive waves of myoblasts, which are the primary fibers mainly formed in E3–E7 and the secondary fibers mainly formed in E8–E16 (Bandman and Rosser, 2000; Picard et al., 2002). Therefore, the number of DMRs was detected to rise dramatically at E17, suggesting that E17 may be a crucial period for chicken embryonic skeletal muscle development. Genes that overlapped with DMR at different times were regarded as DMGs and used for GO analysis. We found that DMGs at E11 was significantly enriched in muscle organ development (GO: 0007517) and regulation of skeletal muscle satellite cell proliferation (GO: 0014842), DMGs at E17 was significantly enriched in actin filament depolymerization (GO: 0030042). These terms are closely related to muscle development, thus, the DMGs at E11 and E17 may have an important role in the fetal phase of muscle development in chicken (Stockdale and Miller, 1987).

In addition, we also found that the methylation levels of the promoter regions of these GO term-related genes exhibited an opposite trend to their expression levels. Many of them are widely reported genes closely related to the growth and development of skeletal muscle, including those that regulates the muscle-specific genes expression, *MYOG* (Yin et al., 2011), promotes the proliferation of muscle precursor cells, *FGF2* (Velleman, 2007), and stimulates muscle cell differentiation, *MEF2D* (Wang et al., 2015), etc. These lower promoter methylation levels may be closely related to the rapid development of skeletal muscles in the middle and late stages of the embryo.

To characterize the correlation between gene methylation and expression levels, we further focused on identifying differentially methylated (DMR-associated) and differentially expressed genes through DNA methylation profile and RNA-seq data. We have obtained 50 model profiles after the STEM analysis of 12,473 differential genes. Among them, we found that profile 48 always showed an obvious enrichment in muscle-related GO terms, such as skeletal muscle tissue development (GO:0007519), muscle cell development (GO:0055001), muscle cell differentiation (GO:0042692), etc. By integrating the methylation levels of genes corresponding to their expression profiles, it is found that their positive and negative relationship in genes were both differentially methylated and expressed. In particular, there are several genes which are strongly correlated with the methylation-caused expression switch.

We chose to study the function of the *CFL2* gene in embryonic skeletal muscle development further. The role of *CFL2* has been studied in cardiac muscle (Vafiadaki et al., 2020) and many types of congenital myopathies (Rosen et al., 2020), but not in chicken skeletal muscle development. Qian et al. (2017) found significant differences in the *CFL2* gene in chicken leg muscle transcriptomes of different ages. In the present study, we proved that *CFL2* acts as a negative regulator of chicken skeletal muscle satellite cell proliferation and can induce cell apoptosis. Furthermore, *CLF2* has been reported to be an essential mediator for myogenic differentiation in C2C12 myoblasts (Mai et al., 2020). These results suggest that *CFL2* is regulated by DNA methylation and participates in muscle development during embryonic stages. Subsequent knockdown and Western blot assay verified the results of our analysis, indicating the reliability of our analysis and the role of *CFL2* in muscle cells proliferation and apoptosis.

In conclusion, we revealed a comprehensive DNA methylome and transcriptome landscape during the embryonic developmental stages in chickens, and identified that *CFL2* gene plays a significant role in regulating SMSCs proliferation and apoptosis. Moreover, these results provide valuable data for future genome and epigenome studies of chicken skeletal muscle and may help reveal the molecular mechanisms of potential economic traits.

## Data Availability Statement

The data in this study has been deposited into BioProject (accession: PRJNA723059, https://www.ncbi.nlm.nih.gov/bioproject/PRJNA723059).

## Ethics Statement

The animal study was reviewed and approved by the Animal Ethics Committee of Sichuan Agricultural University, Chengdu, China.

## Author Contributions

JR performed the experiments, analyzed the data, designed the study, and wrote and reviewed the manuscript. LY and DZ collected the samples. YL and CWY performed the project administration. CLY and JL performed the experiments. HD and XJ analyzed the data. All authors read and approved the final manuscript.

## Conflict of Interest

The authors declare that the research was conducted in the absence of any commercial or financial relationships that could be construed as a potential conflict of interest.

## Footnotes

1 ftp://ftp.ensembl.org/pub/release-101/gtf/gallus_gallus/

2 ftp://ftp.ensembl.org/pub/release-101/fasta/gallus_gallus/dna/Gallus_gallus.GRCg6a.dna.toplevel.fa.gz

3 https://www.cs.cmu.edu/~jernst/stem/#

4 http://www.cytoscape.org/

## Supplementary Material

Supplementary Figure 1 Classification of methylated cytosines at four different developmental stages.

Supplementary Figure 2 Distribution of methylation levels in functional regions.

Supplementary Figure 3 Distribution of DMR gene regions obtained by pairwise comparison of four different developmental stages.

Supplementary Figure 4 Distribution of DMR gene length obtained by pairwise comparison of four different developmental stages.

Supplementary Table 1 Gene Ontology (GO) analysis of PPI network proteins.
