# Supplementary figures and images for: Comparative Analysis of Skeletal Muscle DNA Methylation and Transcriptome of the Chicken Embryo at Different Developmental Stages
Source: Front Physiol. 2021 Jul 2;12:697121. doi: 10.3389/fphys.2021.697121 (PMC8283280; doi:10.3389/fphys.2021.697121)

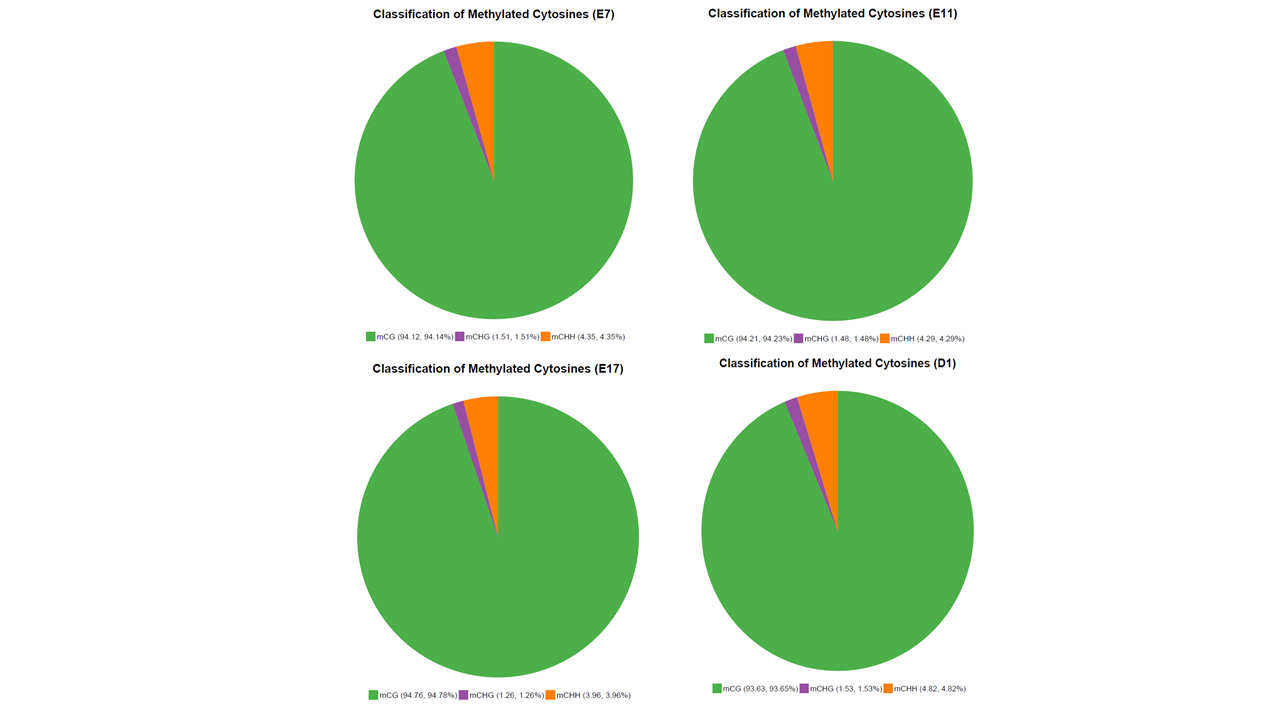

Supplement: Supplementary Figure 1 — Classification of methylated cytosines at four different developmental stages. [file Image_1.TIF]

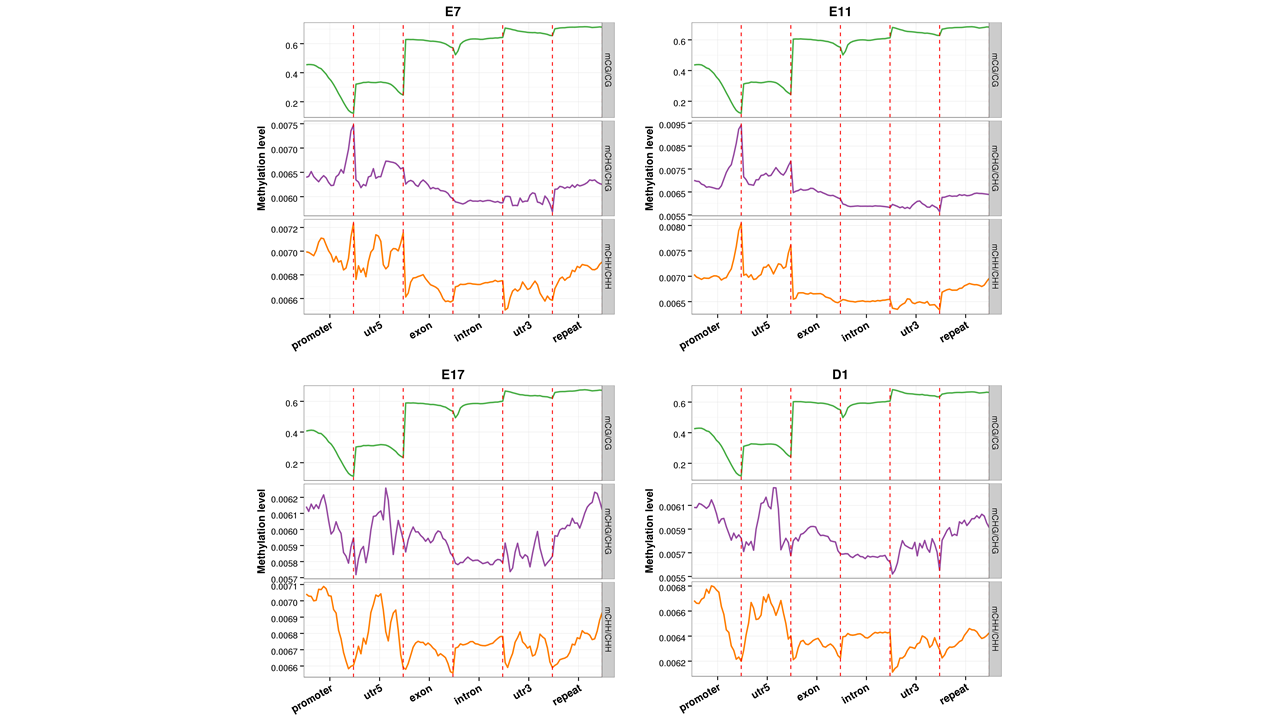

Supplement: Supplementary Figure 2 — Distribution of methylation levels in functional regions. [file Image_2.TIF]

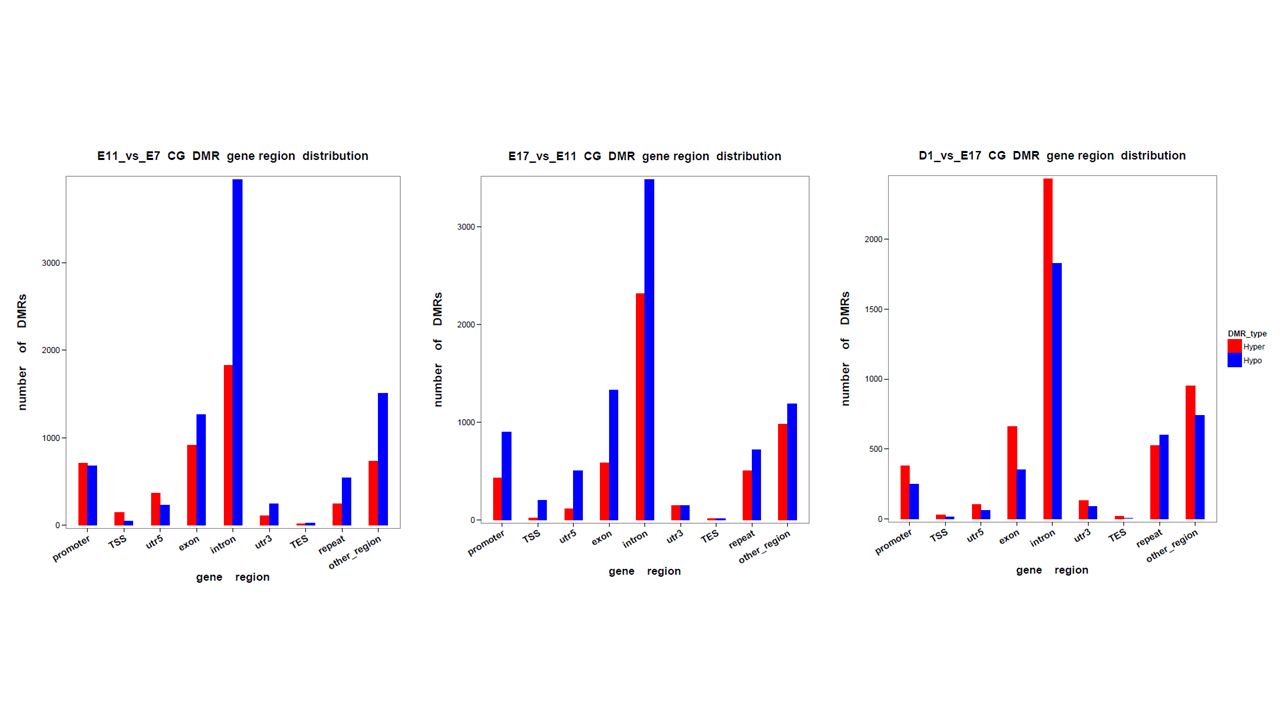

Supplement: Supplementary Figure 3 — Distribution of DMR gene regions obtained by pairwise comparison of four different developmental stages. [file Image_3.TIF]

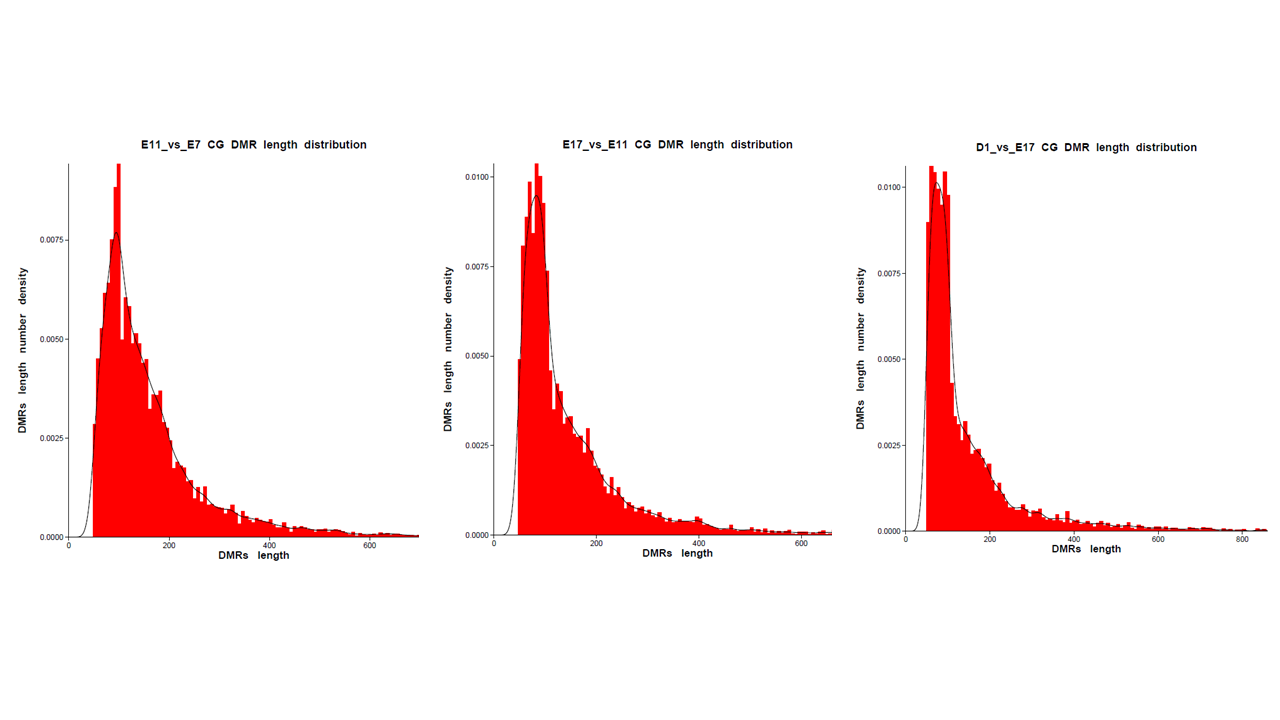

Supplement: Supplementary Figure 4 — Distribution of DMR gene length obtained by pairwise comparison of four different developmental stages. [file Image_4.TIF]
